# Temperature-responsive PCL-PLLA nanofibrous tissue engineering scaffolds with memorized porous microstructure recovery

**DOI:** 10.3389/fdmed.2023.1240397

**Published:** 2023-09-26

**Authors:** Seth M. Woodbury, W. Benton Swanson, Lindsey Douglas, David Niemann, Yuji Mishina

**Affiliations:** 1 Department of Biologic and Materials Science, Division of Prosthodontics, School of Dentistry, University of Michigan, Ann Arbor, MI, United States; 2 Department of Chemistry, College of Literature, Science and the Arts, University of Michigan, Ann Arbor, MI, United States; 3 Department of Physics, College of Literature, Science and the Arts, University of Michigan, Ann Arbor, MI, United States

**Keywords:** tissue engineering, network polymer, biodegradable polymer, scaffold, macropore

## Abstract

Biomaterial scaffolds in tissue engineering facilitate tissue regeneration and integration with the host. Poor healing outcomes arise from lack of cell and tissue infiltration, and ill-fitting interfaces between matrices or grafts, resulting in fibrous tissue formation, inflammation, and resorption. Existing tissue engineering scaffolds struggle to recover from deformation to fit irregularly shaped defects encountered in clinical settings without compromising their mechanical properties and favorable internal architecture. This study introduces a synthetic biomaterial scaffold composed of high molecular weight poly (L-lactic acid) (PLLA) and an interpenetrating network of poly (ε-caprolactone) (PCL), in a composition aiming to address the need for conformal fitting synthetic matrices which retain and recover their advantageous morphologies. The scaffold, known as thermosensitive memorized microstructure (TS-MMS), forms nanofibrous materials with memorized microstructures capable of recovery after deformation, including macropores and nanofibers. TS-MMS nanofibers, with 50–500 nm diameters, are formed via thermally induced phase separation (TIPS) of PLLA after *in situ* polymerization of PCL-diacrylate. A critical partial-melting temperature of TS-MMS at 52°C enables bulk deformation above this temperature, while retaining the nanofibrous and macroporous structures upon cooling to 37°C. Incorporation of drug-loaded poly (lactide-co-glycolide) (PLGA) nanoparticles directly into TS-MMS nanofibers during fabrication allows sustained release of a model drug for up to 40 days. Subcutaneous implantation *in vivo* using LysM-Cre;td-Tomato; Col1eGFP mice demonstrates successful cellularization and integration of deformed/recovered TS-MMS materials, surpassing the limitations of deformed PLLA scaffolds, to facilitate cell and vasculature infiltration requisite for successful bone regeneration. Additionally we demonstrated a method for embedding controlled release vehicles directly into the scaffold nanofibers; controlled release of simvastatin enhances vascularization and tissue maturation. TS-MMS scaffolds offer promising improvements in clinical handling and performance compared to existing biomaterial scaffolds.

## 1. Introduction

Tissue engineering aims to repair and replace tissue lost to trauma or disease (1). Craniofacial bone tissue engineering represents a broad range of compelling clinical needs, including trauma, ridge and sinus augmentation, and tooth extraction socket preservation, with over $190 million spent annually on bone substitute materials alone (2). Particulate bone grafts are packable and fit the irregular shape of these defects well but lack osteoactive capacity and dimensional stability with time, in addition to the risk of immunogenic reaction (3). Biomaterial scaffolds for tissue engineering pose many advantages by taking advantage of endogenous cell sources and the body’s capacity to heal, organizing regeneration in an artificial extracellular matrix (4). However, a significant shortcoming in their clinical adoption is the lack of handling properties that allow for their deformation without irreversibly disrupting their favorable morphology, which we and others have previously elucidated (5–7). In addition, biomaterial scaffolds must come into contact with the bone; ill-fitting interfaces between tissue engineering matrices or grafts and the defect margin lead to poor healing outcomes due to fibrous tissue formation, inflammatory infiltrate, increased risk of infection, and resorption (8). Therefore a biomaterial capable of conformal fitting and volumetric filling of its highly porous internal structure are highly desirable.

Synthetic polyesters such as poly (L-lactic acid) (PLLA), poly (ε-caprolactone) (PCL), and poly (lactide-co-glycolide) (PLGA) are biodegradable, biocompatible materials that are FDA approved for human use, and have been widely studied for a variety of applications (4). PLGA is rapidly degrading but lacks mechanical robustness and is commonly used in nanoparticle applications. PLLA can be synthesized at a high molecular weight, and its crystallinity allows for high tensile and compressive strength. Its degradation is tuned by its molecular weight, porosity, and crystallinity, degrading *in vivo* on the order of 6–9 months (9). The crystallinity of PLLA is suitable for nanofiber formation by its thermally induced phase separation (TIPS) from organic solvent at low temperatures, resulting in fibrillar nanofibers with an average diameter of 50–500 nm, analogous to the collagen extracellular matrix of bone, endorsing its use as a scaffold matrix (10). Nanofibers are well-recognized to promote tissue integration, cell and protein adhesion, and cell proliferation, compared to smooth substrates (11). Interconnected macropores are imparted by a sugar sphere porogen method, allowing for well-controlled pore size and shape (12). Void space and porosity are well-recognized design criteria for scaffold success, particularly to enable cell infiltration, migration, and nutrient/waste exchange (4). We have previously demonstrated the influence of pore size (6), and the biological mechanism by which curvature influences skeletal stem cell fate in craniofacial bone regeneration (5), highlighting their importance.

While PLLA has a high compressive and tensile strength, it is brittle and not amenable to deformation and recovery at the macro (defect), micro (pore), and nano (nanofiber) scales (13). Various groups have described curable resin materials injected into a defect and then polymerized *in situ* within the defect site (14, 15). However, these formulations pose concerns about residual photo-initiators and unreacted monomers causing local toxicity, highly exothermic polymerization, polymerization shrinkage in the defect, and cell death from UV light and heat required for polymerization initiation. Additionally, these materials often lack porosity critical to allow cell infiltration and tissue integration. Compared to PLLA, PCL has longer degradation times, and PCL-based materials cannot form nanofibers or other cell-favorable architecture, limiting their use. However, its low melting temperature and ductility allow for thermally induced deformation without breaking (16, 17).

In the context of bone augmentation, the next generation of biomaterials must account for key processes involved in bone formation and enable therapeutic properties of the biomaterial construct to improve the predictability of outcomes (18). Both intramembranous and endochondral ossification processes heavily rely on the ingrowth of vasculature to supply nutrients, oxygen, and essential signaling factors (19). Therefore, it becomes crucial for biomaterial scaffolds to possess patent pores, *in situ*, that facilitate the infiltration and proliferation of endothelial and mesenchymal cells, ensuring adequate angiogenesis and subsequent bone development. Moreover, the effectiveness of the regeneration strategy is significantly influenced by the precise control of drug delivery (20). Traditional approaches involving nonspecific attachment or adhesion of drugs to the scaffold surface often lead to irregular and uncontrollable release kinetics and premature release, hampering therapeutic efficacy and increasing the risk of adverse effects (21, 22). To overcome these limitations, integrating a drug delivery vehicle inherent to the scaffold’s pores emerges as a promising solution. Such an intrinsic drug delivery system allows for the precise dosing, loading, and release of bioactive agents within the local microenvironment. By providing a sustained and controlled release of growth factors, cytokines, or other therapeutic molecules, this novel approach enhances the potential to modulate cellular responses, promote tissue regeneration, and tailor the healing process according to specific clinical needs.

Herein we have developed a novel thermosensitive memorized microstructure (TS-MMS) tissue engineering scaffold taking advantage of the favorable properties of PLLA and PCL to develop a nanofibrous, macroporous scaffold with internal feature recovery after deformation and nanofiber-embedded drug delivery, enabling conformal fitting of irregularly shaped defects in the surgical setting without compromising its favorable internal structures. We use low molecular weight PCL-DA, a photopolymerizable PCL oligomer, to fabricate interpenetrating copolymer meshes in various PCL-DA/PLLA compositions to determine the boundary conditions for nanofiber formation and thermoresponsive properties. We demonstrate the reversible deformation of TS-MMS scaffolds above a critical temperature of 52°C and assess their ability to facilitate cell and tissue infiltration *in vivo* after their deformation and recovery, demonstrating their advantageous memorized microstructure recovery. This feature allowed us to modify the shape of the scaffolds at 52°C to achieve conformal fitting to the abnormally shaped defects, regaining their original mechanical rigidity upon cooling to physiologic temperature. Acellular TS-MMS scaffolds implanted subcutaneously enable robust vascularization and extracellular matrix deposition, demonstrating a proof of concept for their ability to facilitate robust tissue integration. Additionally we demonstrate the development of a nanofiber-embedded drug delivery platform. This is the first report to our knowledge of a nanofibrous biomaterial capable of shape memory, filling a critical need for off-the-shelf synthetic, biodegradable materials which can be fabricated at scale with favorable clinical handling properties.

## 2. Materials and methods

### 2.1. Materials

Resomer 207S poly (L-lactic acid) was purchased from Evonik. All other reagents were purchased from Sigma Aldrich unless mentioned in the methods below. Reagents were used as received unless otherwise specified.

Polymer synthesis and spectroscopy is described in supplemental methods.

### 2.2. 2D thin film fabrication

A 3 mm stock solution of Irgacure^®^ 2959 in methanol was prepared. A tetrahydrofuran (THF) polymer solution was separated and heated to 62°C until the polymers were dissolved entirely. 3.33% v/v photoinitiator solution was added to the polymer solution and mixed before being rapidly transferred into 3D printed molds (approximately 2–2.5 ml of solution per film mold) and placed in a FisherScientific^®^ UV Crosslinking Chamber (λ = 256 nm) powered at E = 10 J for five minutes. Films were immediately transferred onto flat slabs of dry ice to induce TIPS, then transferred into a −80°C freezer for 48 h. After two days at −80°C, the films were removed from the freezer and placed into an ice bath for 3 h. The resulting films were dried flat for 4 days and stored at −2°C until further use.

Physical, mechanical, and thermal properties were evaluated as described in the supplemental methods.

### 2.3. Shape-memory thermal cycling of films

Films were placed in an 80°C water bath (regulated with digital heating plate) and held underwater for 30 s. After this time, the films were mechanically coiled around a 1 mm diameter metal rod, then transferred into an ice bath. Finally, the locked-structure film was transferred back into an 80°C or 55°C water bath to complete the thermal cycle. Film recovery was filmed using a digital camera. The recorded videos were then analyzed to measure recovery time, *n* > 5 for each group.

### 2.4. Scaffold fabrication

Nanofibrous, macroporous scaffolds are fabricated as described in the literature by our group and others (5, 6, 12). PLLA or TS-MMS polymer solutions were prepared at 62°C. 0.8–1.2 ml of the polymer solution was poured into each mold and subjected to three rapid vacuum cycles to penetrate the sugar template. In the case of TS-MMS: A 3 mm stock solution of Irgacure^®^ 2959 in methanol was prepared. 1.7% v/v ratio photoinitiator stock solution was injected into the polymer solution (170 μl photoinitiator per 10 ml of polymer solution). The solution was quickly stirred, poured into the molds, and vacuum cycled. All constructs underwent UV-induced crosslinking (FisherScientific^®^ UV Crosslinking Chamber, λ = 256 nm) at E = 10 J for 10 min. The resulting scaffolds were immediately transferred to −80°C for 48 h. Scaffolds were transferred to hexane at room temperature on an orbital shaker set at 80 rpm for one day then submerged in distilled water for 1 day. The scaffolds were cut and stored at −80°C until further use.

### 2.5. PLGA nanoparticle fabrication

Nanoparticles were prepared by a w/o/w double emulsion method (23). Details of their fabrication for each composition are in the supplemental methods.

### 2.6. Fabrication of nanoparticle-embedded nanofibers

15 mg of PLGA nanoparticles were constituted in 1 ml hexane containing 2 μl of Span80 surfactant, vortexed, and mixed with sugar spheres to facilitate reversible hydrophilicity-mediated surface adhesion. As described above, a sugar sphere template was formed from the nanoparticle-coated sugar spheres, and polymer casting was performed according to the same protocol. Evaluation of drug-release kinetics is described in the supplemental methods.

### 2.7. Sterilization of biomaterial constructs

First, constructs were sterilized by ethylene oxide gas according to the manufacturer’s protocol (Anpro). Secondly, scaffolds were washed with 70% ethanol for 30 min, followed by PBS in a biosafety cabinet immediately prior to implantation.

### 2.8. Subcutaneous implantation in mice

Nanofibrous scaffolds (8 mm diameter × 1.5 mm height) as described above were implanted subcutaneously into LysM-Cre; td-tomato;Col1eGFP male mice aged 10–12 weeks old. All animal procedures followed a protocol approved by the University of Michigan Institutional Animal Care and Use Committee (IACUC) (Animal Study Protocol #PRO00009613 and #PRO00011263). On the day of implantation, mice were anesthetized via isoflurane inhalation, shaved, and a midsagittal incision was made on the dorsal surface of each mouse. On each side of the midline, two pockets were made by blunt dissection such that four acellular scaffolds were implanted into each mouse. The incision was closed with sutures, and mice were given post-operative analgesic medication (carprofen) to manage pain for 24 h or longer as needed. Mice were monitored closely and showed no adverse signs. After 4-weeks, mice were euthanized by inhalation of CO_2_ and bilateral pneumothorax. Constructs were carefully explanted and fixed in 4% paraformaldehyde for 24 h. Histologic assessment is described in the supplemental methods.

### 2.9. Statistical methods

All data are reported as mean ± standard deviation and represent a minimum sample size of *n* > 3. Statistical analysis was carried out in GraphPad Prism v9. Student’s *t*-test was used to determine the statistical significance of observed values between experimental groups where *p* < 0.05 was considered significant. All graphics note significance as: * *p* < 0.05, ** *p* < 0.01, ***p < 0.001, ****p < 0.0001.

## 3. Results

### 3.1. Thermosensitive nanofiber-forming biomaterial optimization

**FIGURE 1:**
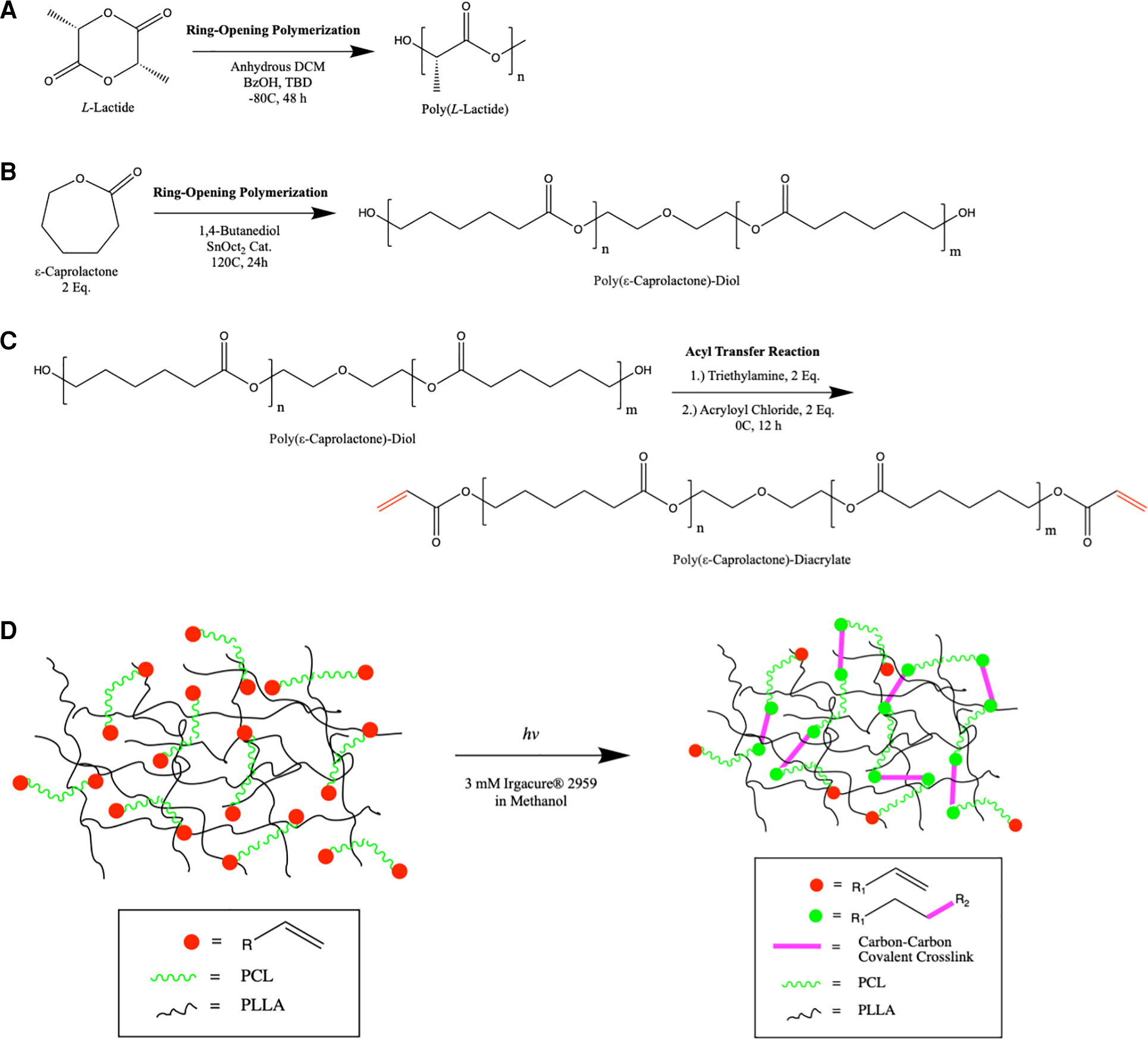
Schematic overview of the synthesis of PLLA (A) and PCL (B) by ring opening metastasis polymerization. Bifunctional PCL is modified with acryloyl chloride at its termini by nucleophilic substitution (C) In situ polymerization of PCL-DA within the high molecular weight PLLA mesh is shown (D), highlighting the formation of new covalent bonds in the memorized microstructure.

We hypothesized that a thermosensitive biomaterial with memorized microstructure may be fabricated by the in-situ polymerization of a thermosensitive polyester mesh, PCL, within a rigid high molecular weight PLLA matrix. PLLA and PCL are synthesized by ring-opening metastasis polymerization (Figures 1A,B). The terminal functional groups of PCL are acrylate-modified by nucleophilic substitution with acryloyl chloride (Figure 1C). Low molecular weight PCL-DA oligomers (2–20 kDa) are dissolved in solution with high molecular weight PLLA (150 kDa), cast to a specified shape, and subjected to photoinitiated radical polymerization causing the chain extension PCL-DA in the cast shape, termed “memorized structure” (Figure 1D).

**FIGURE 2:**
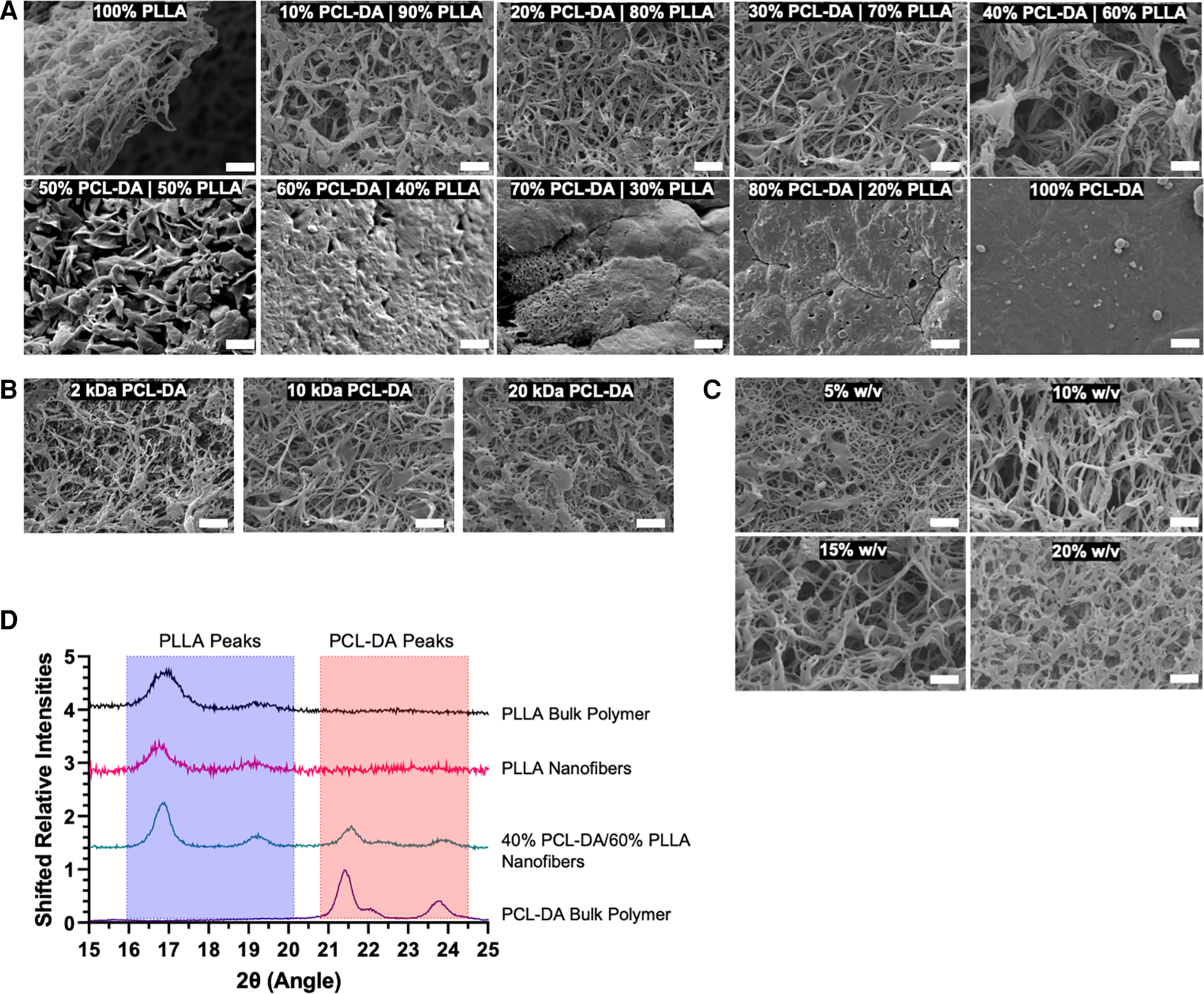
Scanning electron micrographs (SEM, 5,000×) are used to evaluate nanofiber formation as a result of thermally induced phase separation in 2D thin films by variable PCL-DA/PLLA composition (A), scale = 2 um), PCL-DA molecular weight (B), scale = 2 um) and total material % w/v in solution (C), scale = 2 um). In addition, small angle x-ray scattering spectra demonstrate the maintenance of bulk polymer crystallinity during material fabrication (D).

To determine the ability of this semi-interpenetrating polymer mesh to form nanofibers, we synthesized two-dimensional thin films with varying compositions and evaluated nanofiber formation by scanning electron microscopy (Figure 2A). Nanofibers formed at 0% w/w PCL-DA up to 40% w/w PCL-DA, where the remaining composition is PLLA, are indistinguishable. A platelet morphology is observed at 50% PCL-DA/50% PLLA. Beyond 60% PCL-DA incorporation, nanofiber morphology is lost due to the amorphous structure of PCL-DA. At 40% PCL-DA/60% PLLA, the molecular weight of PCL-DA does not affect nanofiber formation when PLLA molecular weight is held constant (Figure 2B). Similarly, nanofibers are formed at 40% PCL-DA 10 kDa/60% PLLA up to 20% total material w/v when cast from solution (Figure 2C). Small angle x-ray scattering demonstrates the maintenance of PLLA crystallinity with characteristic peaks (24) at 2θ = 17°, 19°, which are preserved when fabricated as nanofibers and when combined with PCL-DA at up to 40% PCL-DA/60% PLLA (Figure 2D).

### 3.2. Evaluation of shape memory capacity for memorized microstructure

**FIGURE 3:**
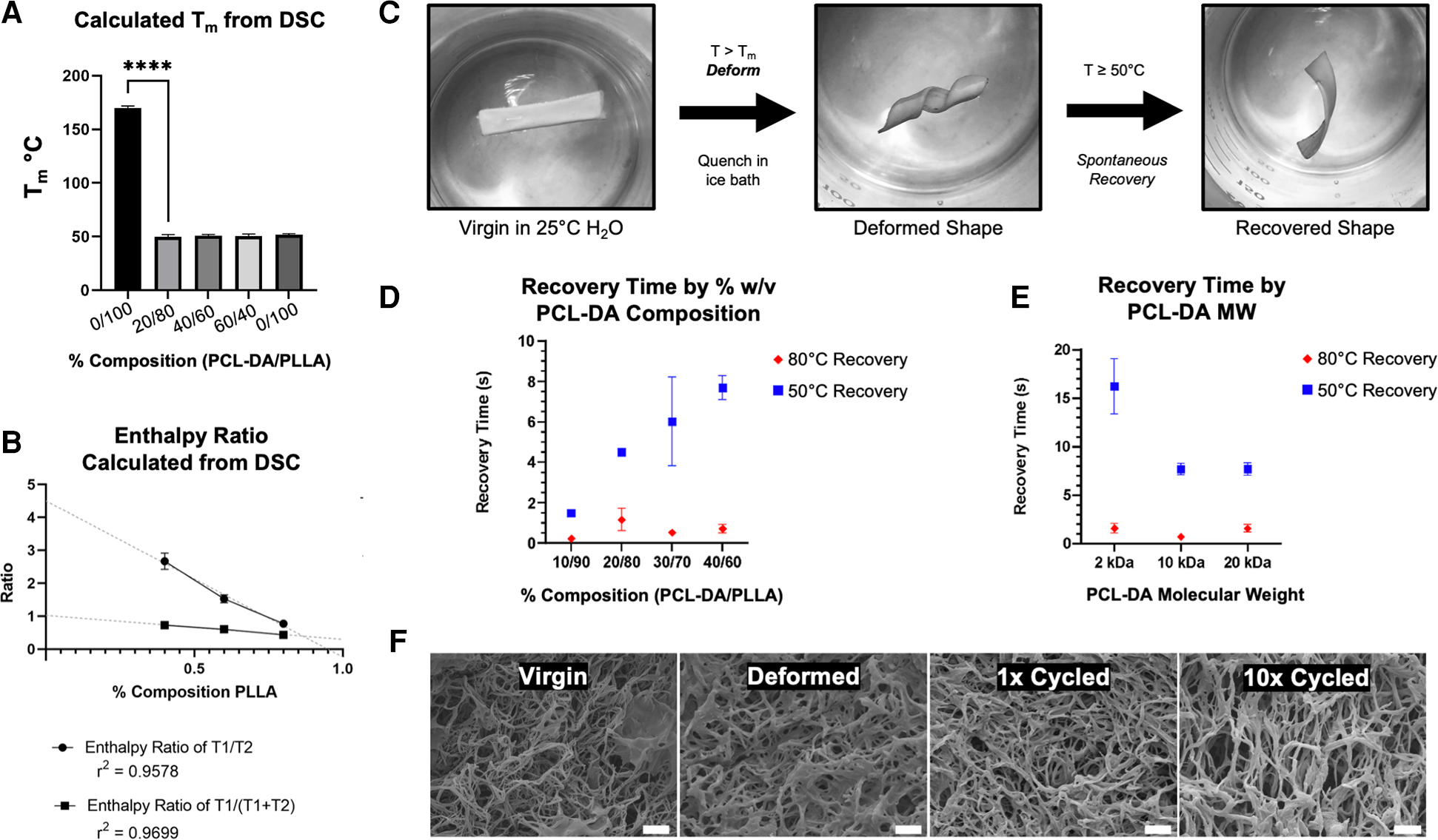
Melting temperature (T_m_) is calculated from dynamic scanning calorimetry (DSC) (A) and enthalpy ratio is calculated by the ratio of T_1_ (52°C, PCL-DA) and T_2_ (165–172°C, PLLA) peaks (B) shape memory capacity is evaluated by the deformation and recovery of 2D thin film materials, as shown (C), and measured as a function of PCL-DA composition (D) and PCL-DA molecular weight (E) SEM evaluates nanofiber morphology maintenance as a cycling function (F, 5,000×, scale = 2 um).

Dynamic scanning calorimetry demonstrates a marked reduction in T_1_ melting temperature (T_m_) from 165°C to 52°C upon incorporating PCL-DA (Figure 3A). It remains the same irrespective of the relative composition of PCL-DA, comparable to PCL-DA alone. Where T_1_ is 52°C, corresponding to the melting temperature of PCL-DA, a T_2_ exists at 165°C, corresponding to the PLLA component (data not shown). Incorporating PCL-DA introduces a partial melting temperature to the PLLA matrix at a significantly lower temperature (T_1_). The enthalpy ratio of PCL-DA to PLLA (T_1_/T_2_) and PCL-DA to the total material [T_1_/(T_1_+ T_2_)] is inversely proportional to the relative PLLA contribution to the entire material composition (Figure 3B), enabling stoichiometric titration of temperature-sensitivity. We confirmed the two-component nature of our system and determined its degradation kinetics in comparison to PLLA and PCL-DA by thermogravimetric analysis (25) (TGA, Supplementary Figure S1).

We hypothesized that materials were readily deformable at temperatures above T_m_, and recoverable to their original 3D morphology at the macro, micro, and nano scales. We developed an assay to determine shape recovery, as shown in Figure 3C, where virgin materials were subjected to a warm water bath at T > T_m_ for 1 min, then deformed and quenched in an ice bath to prevent recovery. Quenched materials are reintroduced to warm water baths at either 80°C or 50°C, and their spontaneous recovery is recorded using a digital camera for quantitative video frame analysis. At 80°C and 50°C, 100% PLLA materials are neither deformable without yielding nor recoverable, as expected. At 80°C (T > T_m_), all TS-MMS compositions, as low as 10% PCL-DA, exhibit rapid recovery; at 50°C (T ⩬ T_m_), the recovery time is directly proportional to the relative composition by PCL-DA within the range of compositions capable of forming biomimetic nanofibers (Figure 3D). At 40% PCL-DA/60% PLLA, scaffolds with 2 kDa PCL-DA recovered more slowly than 10 kDa and 20 kDa at 50° C; no difference was observed at 80°C where all formulations recovered rapidly (Figure 3E). After deformation and recovery, TS-MMS nanofibers are indistinguishable from virgin material. Even after ten cycles of deformation and recovery, nanofibers remain indistinguishable (Figure 3F). For all subsequent fabrication, 40% PCL-DA (10 kDa)/60% PLLA was used to maximize thermosensitive shape recovery in subsequent validation experiments.

### 3.3. Fabrication of nanofibrous, macroporous scaffolds with thermosensitive memorized microstructure

**FIGURE 4:**
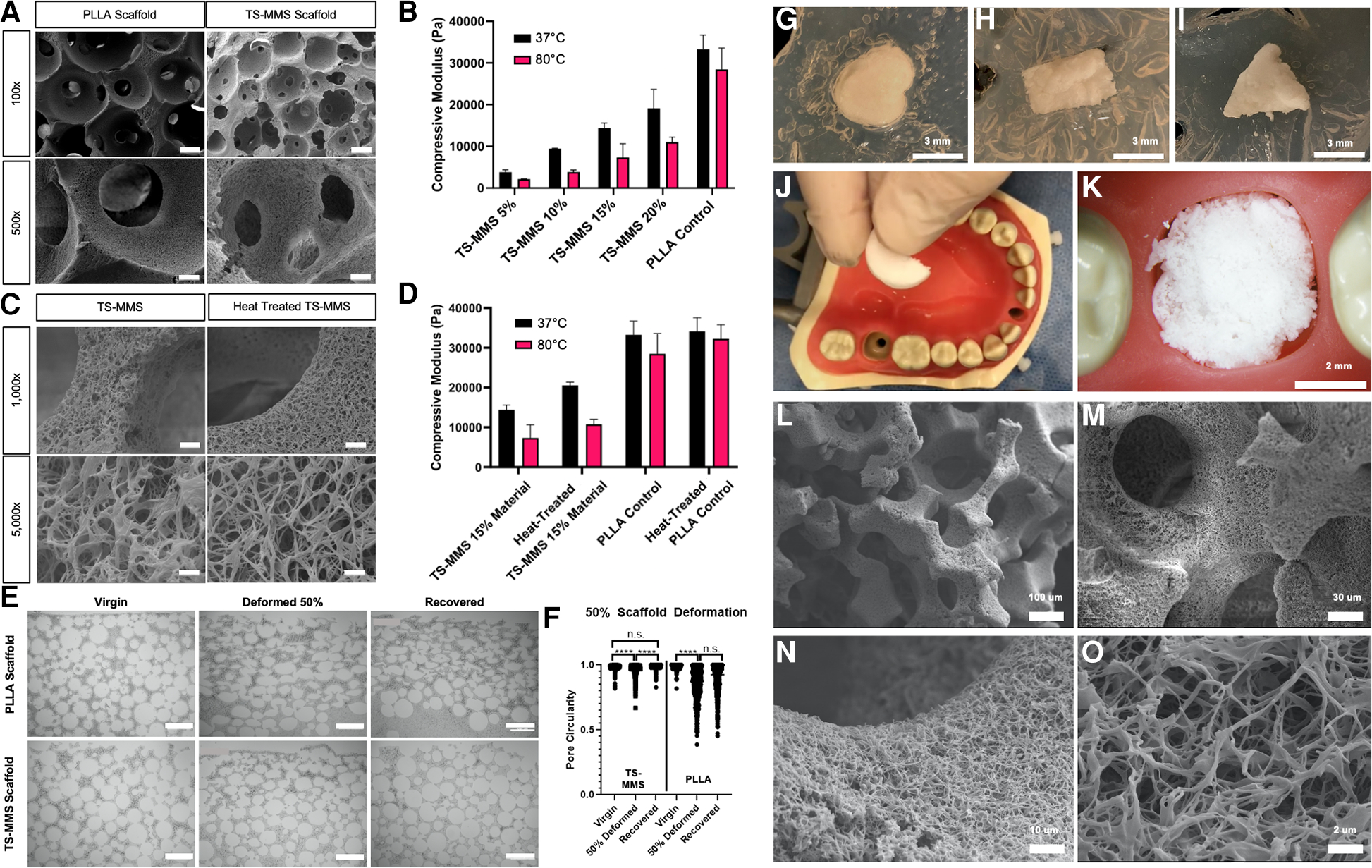
SEM micrographs of scaffolds fabricated from PLLA and TS-MMS (15% w/v, 40/60 PCL-DA/PLLA) at 100× (scale = 100 um) and 500× (scale = 20 um) (A) compressive moduli of scaffolds as a function of material % w/v composition is shown as a function of temperature (B) SEM micrographs of virgin and heat-treated TS-MMS scaffolds show no difference in nanofiber morphology at 15% w/v (C) heat treatment improves the compressive modulus of 15% w/v TS-MMS scaffold at 37°C (D) scaffolds were deformed in a mechanical tester, then recovered. At each step, histologic sections were examined (E) and pore circularity was quantified by image analysis (F) 5 mm round scaffolds are heated and deformed into various irregular shapes cut from agar in a petri dish (G–I). A semi-circle-shaped TS-MMS scaffold was heated and placed into a mannequin typodont tooth socket as a proof of concept (J,K). SEM evaluation of its morphology after deformation demonstrates maintenance of interconnected spherical macropores (L,M) and nanofibrous surface texture (N,O).

Nanofibrous, macroporous scaffolds were fabricated by a sugar sphere porogen method (12), *in situ* polymerization of the TS-MMS, and thermally induced phase separation yielding scaffolds that are indistinguishable from PLLA control scaffolds by SEM (Figure 4A). The compressive modulus of TS-MMS scaffolds varies as a function of total material weight percent as fabricated and is less than PLLA at the same composition (15% w/v, Figure 4B). The compressive moduli of TS-MMS scaffolds, but not PLLA, decrease significantly at T > T_m_ (80°C). We hypothesized that heat treatment of the TS-MMS scaffolds would improve their mechanical properties by annealing PCL-DA within the confines of the crosslinked matrix to adopt minimum energy organization at a temperature between T_1_ and T_2_, such that PLLA does not melt. TS-MMS scaffolds were heat treated at 75°C for 60 min, then cooled to room temperature. SEM micrographs show no adverse effect of heat treatment on nanofiber formation (Figure 4C), and a marked increase in compressive modulus of the 15% w/v TS-MMS scaffold at 37°C (Figure 4D). No significant change was noted in the mechanical properties of virgin versus heat-treated PLLA scaffolds, attributing the transformation to the PCL component.

We hypothesized that TS-MMS fabrication and *in situ*, polymerization of PCL-DA within the sugar sphere template would enable memorization of spherical macropore architecture and their recovery. 15 mm round TS-MMS and PLLA scaffolds were heated at 55°C for 5 min, then deformed by compressing in a mechanical tester to 50% of their height (3.0 mm, compressed by 1.5 mm) and quenched at 0°C in the deformed state. Then, scaffolds were placed in a 55°C water bath to recover for 5 min and cooled to room temperature. Virgin, deformed, and recovered scaffolds from PLLA and TS-MMS were subjected to bulk serial sectioning and histologic analysis for macropore circularity (Figures 4E,F). Image analysis of 40% PCL-DA/60% PLLA scaffolds demonstrates significant pore deformation, and recovery insignificantly different from the virgin scaffold.

On the other hand, 100% PLLA scaffolds are deformed and fail to recover, with deformed and recovered pore circularity not significantly different (Figure 4F). Notably, there was no significant difference between the macropore circularity of the virgin and recovered TS-MMS scaffold, suggesting that the macropores were not damaged during the thermosensitive cycle.

As proof of principle, circular TS-MMS scaffolds were deformed to fit irregular shapes cut from agar (Figures 4G–I). We demonstrated using a semi-circle TS-MMS scaffold to fit a tooth extraction socket defect in a plastic typodont (Figures 4J,K). SEM micrographs show maintenance of the internal geometries critical to favorable regeneration outcomes—interconnected, spherical macropores (Figures 4L,M) and nanofibrous surface texture (Figures 4N,O).

### 3.4. Nanofiber-embedded controlled delivery

**FIGURE 5:**
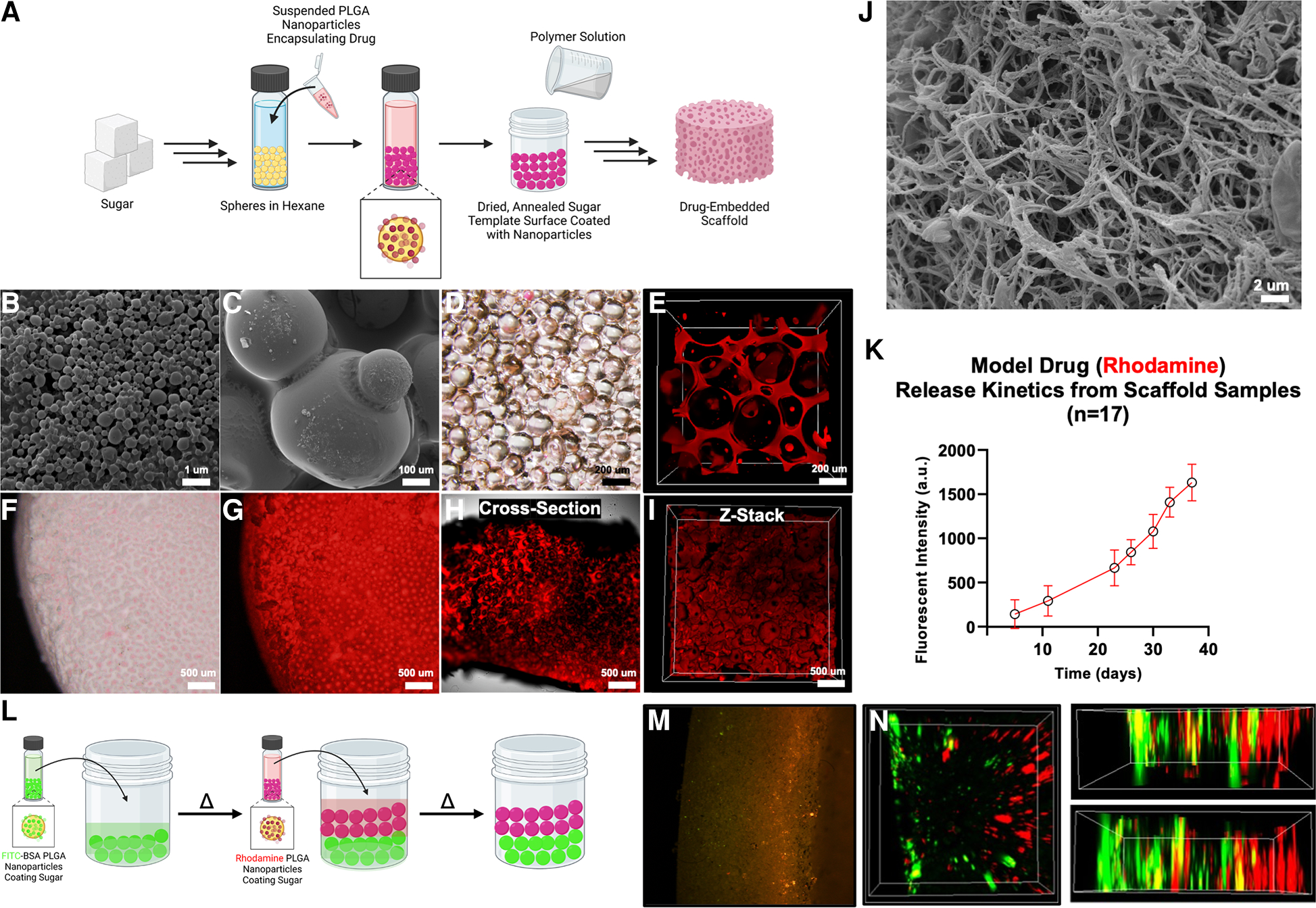
PLGA nanoparticles containing rhodamine are incorporated with the sugar sphere template as shown schematically in (A) PLGA spheres (SEM) (B) are nonspecifically adhered to sugar spheres (SEM) (C), allowing for their uniform incorporation (bright field microscope, (D) 3D confocal laser microscope, (E) the resulting scaffold has uniform functionalization with nanoparticles as shown by bright field (F), fluorescence (G), and confocal laser microscopy (H,I). SEM micrographs demonstrate the incorporation of nanoparticles directly into the nanofiber during thermally induced phase separation (J). Release kinetics of rhodamine from the scaffold demonstrate sustained release kinetics over time (K). Regions of the scaffold may be functionalized with different drugs, for example FITC-BSA and rhodamine, as shown schematically in (L) and by fluorescence (M) and confocal laser microscopy (N), as proof of principle.

Previous attempts to functionalize PLLA scaffolds rely on surface adhesion of nanoparticles after scaffold fabrication (21, 23). We hypothesized that it was possible, and more favorable, to introduce nanoparticles at the initial fabrication step, relying on the nonspecific hydrophilic interaction between poly (lactide-co-glycolide) (PLGA) and D-fructose sugar spheres (Figure 5A). PLGA nanoparticles (NP, d_avg_ = 200 nm) were fabricated by a water-in-oil-in-water double emulsion, as described in the literature (26), containing Rhodamine B (RhB), a small molecule model drug (Figure 5B). RhB-NP was combined with sugar spheres in hexane (Figure 5C) to fabricate the sugar sphere template (dried, Figure 5D). Confocal laser microscopy demonstrates uniform functionalization of the sugar spheres and interstitial spaces with fluorescent rhodamine signal (Figure 5E). After fabrication, the resulting TS-MMS scaffold is uniformly functionalized with rhodamine throughout its bulk (Figures 5F–I). SEM micrographs demonstrate the physical incorporation of RhB-NPs into the nanofibers (Figure 5J), allowing for sustained, controlled drug release over 40 days as the nanofibers degrade (Figure 5K). We then hypothesized that patterning drugs or other inductive signals within the scaffold may be possible by layering NP-functionalized sugar spheres in the scaffold template (Figure 5L), using FITC-BSA-NP, a protein model drug, and RhB-NP drug delivery vectors. Two distinct zones within the dried sugar template are visualized by fluorescence microscopy corresponding to FITC-BSA-NP and RhB-NP (Figure 5M). The resulting scaffold is imaged in three dimensions by confocal laser microscopy demonstrating robust, spatially discrete functionalization (Figure 5N).

### 3.5. TS-MMS macroporous scaffolds enable cell and tissue integration following deformation

**FIGURE 6:**
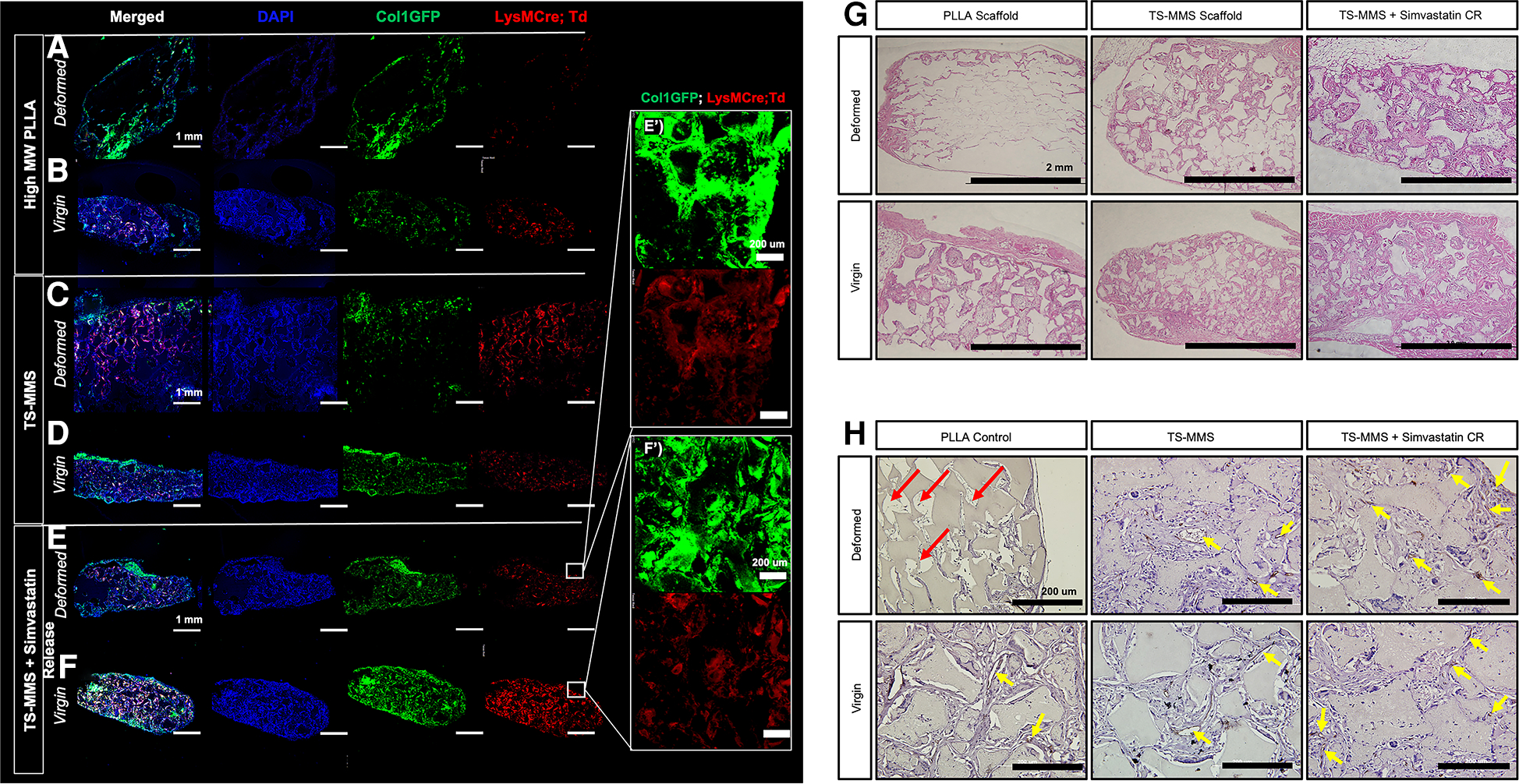
TS-MMS scaffolds were implanted subcutaneously in lysM-Cre;td-tomato;Col1eGFP mice for four weeks, then explanted for histologic analysis. Confocal laser microscopy (A–F) is used to assess cellularity and cellular composition. In addition, Hematoxylin and eosin-stained sections are used to evaluate cellularity (G) and immunohistochemistry for CD31 is used to determine vascularization (H) Red arrows highlight avascularity; yellow arrows highlight CD31 positive signal (brown).

We sought to validate the hypothesis that TS-MMS pore recovery, following deformation, facilitates cell and vasculature infiltration equivalent to a virgin scaffold, compared to a PLLA control. PLLA, TS-MMS, and TS-MMS scaffolds with simvastatin-loaded nanoparticles were fabricated; scaffolds were deformed by 50% mechanical deformation followed by recovery, or left virgin as a control, before subcutaneous implantation in LysM-Cre;td-Tomato;Col1eGFP mice for four weeks. GFP marks cells undergoing osteogenic differentiation. RFP marks cells from the myeloid lineage, including monocytes, macrophages, and osteoclasts. After four weeks, deformed PLLA scaffolds showed negligible cell infiltration into the bulk of the scaffold beyond cell attachment at the perimeter (Figure 6A) compared to their virgin counterparts (Figure 6B), which are well cellularized based on DAPI signal and have both GFP-positive and FRP-positive cells uniformly throughout the scaffold. On the other hand, both deformed (Figure 6C) and virgin (Figure 6D) TS-MMS scaffolds are well cellularized (DAPI) throughout the construct and demonstrate osteogenic potential (GFP) as well as innate immune cell infiltration (RFP). TS-MMS scaffolds with controlled release of simvastatin (27) (Figures 6E,F) facilitated robust cell infiltration and tissue maturation based on total GFP-positive and RFP-positive signals. Hematoxylin and eosin staining confirms the same trend in cellularity observed by DAPI (Figure 6G) where deformed TS-MMS scaffolds (±simvastatin release) are much more well-cellularized than deformed PLLA scaffolds with occluded macropores. No gross fibrous tissue encapsulation or host rejection was observed in all cases. CD31 immunohistochemistry highlights the avascularity of deformed PLLA scaffolds (red arrow, Figure 6H) compared to robustly cellularized, deformed TS-MMS scaffolds (yellow arrows) compared to virgin controls. As anticipated, controlled release of simvastatin augments blood vessel number and size.

## 4. Discussion

Herein we have described synthesizing and fabricating a novel spell out TS-MMS tissue engineering scaffold from biodegradable polymers which maintains the patency of spherical macropores after clinical handling. Macropore patency is critical to facilitate cell, vasculature, and tissue infiltration with the scaffold (4, 6). PCL has broadly been explored as a biomaterial due to its biocompatibility and relatively low melting temperature. Still, its use as a major component in tissue engineering applications has been stunted because of its inferior mechanical properties and long degradation time (16). Our TS-MMS material takes advantage of the advantageous properties of PCL while introducing a nanofibrous surface morphology, and degradation time more similar to that of PLLA.

Biomaterials with robust macroscale shape memory capacity have been made from PCL meshes (17); our strategy aimed to maintain the individual advantageous molecular properties of PLLA (i.e., capacity for TIPS) and PCL (i.e., thermal sensitive properties) in a homogenous composite material. Differential scanning calorimetry demonstrated that the polymers are at least partially immiscible, confirmed by TGA based on the difference in onset temperature between PCL and TS-MMS, but not PLLA and TS-MMS (28). Previously, PLLA copolymers have been demonstrated to form nanofibers under specific compositions and conditions (29). However, this is the first report of PLLA composites capable of developing uniformly nanofibrous matrices with thermosensitive properties. This strategy was considered advantageous over an interconnected polymer network because it provides macromolecular freedom of rearrangement by the PCL-DA interpenetrating within the PLLA matrix (30). Furthermore, nanofiber formation depends on the crystallinity of PLLA, which would not be possible in an statistical copolymer polymer network of PLLA and PCL (31). Finally, our strategy overcomes concerns of the long degradation time of pure PCL matrices; the high degree of porosity and resulting high surface area enables uniform bulk degradation on a time scale most similar to PLLA.

TS-MMS memorized microstructure recovery demonstrates the role of PCL-DA as a chaperone polymer within the total polymer matrix, guiding the crystalline PLLA chains through bulk deformation events. When T > T_1_ (52°C), PCL-DA segments are mobile within the confines of the PLLA chains and act as chaperones to facilitate the mechanical deformation of PLLA nanofibers rather than yielding and enable recovery. A longer recovery duration concomitant with greater PCL-DA content suggests that a greater degree of polymer network rearrangement and relaxation (i.e., shape memory) occurs, assuming a constant speed for molecular rearrangement at a given temperature. Importantly, nanofibers are maintained throughout the deformation and recovery event, repeated for multiple cycles.

The TS-MMS scaffold biomaterial is irradiated to initiate the *in situ* polymerization of PCL-DA around sugar spheres after it is cast to memorize the microstructure of the scaffolds by forming molecular netpoints, then photoinitiator is leached along with the sugar porogen before implantation. Heat treatment allows the PCL component to adopt a lower energy geometric conformation from minor physical rearrangement to reduce the internal stress of the TS-MMS scaffold, resulting in a higher compressive modulus (32). TS-MMS scaffolds are less deformed than PLLA likely because of the PCL component, which imparts a ductility that prevents irreversible deformation and enables resiliency to the macropores even without thermosensitive activation.

Previous attempts at embedding drug delivery systems into PLLA scaffolds have relied on nonspecific surface adhesion due to the inertness of PLLA (21, 23). These methods functionalize the surface of the scaffold rather than the bulk, which is particularly important for scaling the platform and ensuring uniform functionalization throughout the defect site. Additionally, particles nonspecifically adhered will likely wash off the scaffold during pre-implantation soaking and mechanical deformation events. Herein we developed a new method for a nanofiber-embedded drug delivery system during the TIPS process, which improves the durability and consistency of the drug delivery system.

In vivo, we demonstrated that TS-MMS scaffold macropores maintain their ability to facilitate cell and tissue ingrowth after deformation and recovery, comparable to virgin PLLA and TS-MMS controls. The subcutaneous implantation model was chosen specifically to address whether host cells and blood vessels can infiltrate the scaffold after its clinical deformation and spontaneous recovery. Cell infiltration from the host is a critical first step toward regeneration, mainly when relying on endogenous cell sources to populate the scaffold. Previously we demonstrated that macropores of sufficiently large size (>250 um diameter) are required for vascularization and osteogenic differentiation (6); here, we present a method to maintain the patency of macropores during clinical handling. Bone is a highly vascularized tissue; vasculature ingrowth and maturation is a critical step in engineered bone tissue formation (19). Tissue substitutes such as grafts are inherently avascular; the robust vascularization of implanted scaffolds is one significant advantage. Vascularization is also critical for establishing and is regulated by resident innate immune cells, demonstrated by RFP-marked LysM-derived cells. This inflammatory infiltrate supports angiogenesis and tissue remodeling towards host integration and engineered tissue maturation (33). Myeloid progenitors differentiate into osteoclasts, critical to remodeling and turnover. In bone regeneration, non-resorbing preosteoclasts secrete PDGF-BB to recruit endothelial cells and osteoblast precursors, coupling angiogenesis with osteogenesis simultaneously with promoting endothelial endomucin-expression characteristic of Type H blood vessels (34, 35). Therefore, it is an ideal candidate for sustained, controlled release locally. Simvastatin, a common cholesterol-lowering inhibitor of hepatic hydroxymethylglutaryl coenzyme A, has been demonstrated as a potential osteoinductive drug by promoting VEGF and FGF-2 expression at sufficiently low, but not high, sustained doses (36). Statins also increase osteoblast differentiation by increasing BMP-2 expression (37), explaining the increased Col1eGFP (osteogenic) signal in these samples. In the future, immune profiling of the innate immune niche within engineered bone tissue, correlated to histologic tissue maturity and remodeling, may be of significant interest. In the context of craniofacial bone tissue engineering, clinically available biomaterials are manufactured to a standard size and shape, used to fill an irregularly shaped defect as it presents in the clinic. Materials which are capable of conformal fitting and volumetric filling during their placement in the clinical setting, like the TS-MMS scaffold reported here, would allow for rapid cellularization and vascularization, ultimately enabling more predictable osseous wound healing.

## 5. Conclusion

In conclusion, we developed a materials formulation for successfully fabricating and optimizing a thermosensitive nanofiber-forming biomaterial scaffold with memorized microstructure at the macropore and nanofiber scale. A semi-interpenetrating polymer mesh composed of a combination of PLLA and PCL-DA requires a minimum composition of high molecular weight crystalline PLLA to form nanofibers by TIPS. Its shape memory capacity is imparted by a PCL-DA chaperone network polymerized *in situ*, imparting thermosensitive properties. The nanofiber morphology was maintained up to 40% PCL-DA incorporation, beyond which it was lost due to the amorphous structure of PCL-DA. The thermosensitive shape memory capacity of the material was evaluated, and it was found that the recovery time of the deformed scaffolds was directly proportional to the relative composition of PCL-DA. Heat treatment of the scaffolds improved their mechanical properties, and the macropore architecture was successfully recovered after deformation *in vitro*. We also demonstrated a novel method for incorporating nanoparticles into the nanofibers, allowing controlled drug delivery. In vivo, TS-MMS scaffolds, but not PLLA scaffolds, facilitate cell and tissue integration following deformation by conforming to the defect shape while recovering their favorable internal macroporous architecture, which is favorable for robust vascularization and bone formation. This novel TS-MMS scaffold holds promise for craniofacial tissue engineering applications due to its thermosensitivity and ability to recover from clinical deformation to fit irregularly shaped defects.

## Supplementary Material

Supplementary Materials

Supplementary Figure 1

## Data Availability

The original contributions presented in the study are included in the article/Supplementary Material, further inquiries can be directed to the corresponding authors.
