# Supplementary material for: Temperature-responsive PCL-PLLA nanofibrous tissue engineering scaffolds with memorized porous microstructure recovery
Source: Front Dent Med. Author manuscript; Available in PMC 2024 Apr 11. (PMC11008614; doi:10.3389/fdmed.2023.1240397)
Supplement: Supplementary Materials [file NIHMS1954334-supplement-Supplementary_Materials.docx]

**Supplementary Materials**

**Supplementary Figure 1**. The lifetime kinetics of TS-MMS scaffolds was assessed by thermogravimetric analysis (A). A plot of dW/dT as a function of temperature demonstrates that onset temperature of TS-MMS is similar to PLLA, with a second small peak at the same temperature of PCL-DA, providing a proxy for its degradation (B).

**Supplemental Methods:**

**Synthesis of Poly-ε-Caprolactone** (PCL-diol): In the preparation of poly ε-caprolactone (MW = ~10 kDa), 10 mL of ε-caprolactone monomer was added into a 50 mL round-bottom flask along with 89 µL of 1,4-butanediol (1 mol eq). 6.5 µL (0.01 mol eq) of tin(II) 2-ethylhexanoate (Sn(Oct)2) was added to a round-bottom flask with magnetic stir bar, and stirred at a low speed under vacuum. The mixture was heated to 120°C and maintained for 12 hours resulting in a highly viscous solution. After cooling, the solid was redissolved in dichloromethane (DCM) and precipitated into 300 mL of methanol (~5x volume) at 0°C, yielding a white solid, which is concentrated. Precipitation is repeated three times to remove unreacted monomer and catalyst. The resulting solid was dried in a vacuum chamber for two days and stored at -20C. Gel permeation chromatography (GPC) in tetrahydrofuran (THF) solvent assessed molecular weight. Molecular characterization is performed by nuclear magnetic resonance spectroscopy (NMR, CDCl3): 𝝳 = m, 1.48 (2H, CH_2_); m,1.78 ppm (4H, CH_2_); q, 2.44 ppm (2H, CH_2_); d, 4.17 ppm (2H, CH_2_).

**Synthesis of Poly-ε-Caprolactone Diacrylate** (PCL-DA): 5 g of PCL-diol (MWavg = 10.47 kDa) was dissolved in a minimum volume of anhydrous DCM. 135 µL of triethylamine (TEA, 2 mol eq.) was added with stirring at moderate speed at 0°C. After 15 minutes, 78 µL of acryloyl chloride (AC) was added dropwise into the solution over 10 minutes. The reaction was left to proceed overnight, warming to room temperature.The resulting reaction mixture was precipitated into 300 mL of methanol (~5x vol) at 0°C three times to remove unreacted AC and TEA. The concentrated solid was dried in a vacuum chamber for two days and stored at -20C. End-group functionalization was confirmed by NMR (CDCl3): 𝝳 = m, 1.48 ppm (2H, CH_2_); m,1.78 ppm (4H, CH_2_); q, 2.44 ppm (2H, CH_2_); d, 4.17 ppm (2H, CH_2_); dd, 5.8 ppm (CH); m, 6.15 ppm (CH); dd, 6.44 ppm (CH).

**Synthesis of Poly Lactic-co-Glycolic Acid (PLGA):** L-lactide (7.6 mmol, 1.10 g, 1 mol eq), and glycolide (7.6 mmol, 0.88 g, 1 mol eq) and benzyl alcohol (initiator, 0.2 mmol, 208 µL, 0.027 mol eq) were combined and heated at 120°C under vacuum with moderate stirring. Next, 100 µL of Sn(Oct)2 (catalyst, 0.001 mol eq) was injected. After twelve hours, the reaction was opened to air and cooled. A minimum volume of DCM was used to dissolve this solid completely, and the resulting solution was precipitated into 300 mL of -20C methanol (10x vol eq), resulting in a white solid, and repeated three times. The solid was allowed to dry for two days in a vacuum chamber and stored at -20C. Chemical identity and the lactide-glycolide ratio was confirmed by NMR (CDCl3): 𝝳 = m, 4.75 ppm (2H, CH_2_). GPC determined molecular weight (9.89 kDa)

**Nuclear Magnetic Resonance (NMR) Spectroscopy**: ^1^H spectra were recorded with a Varian MR400 spectrometer operating at 400 MHz and room temperature. Spectral analysis is done in MestReNova (Version 12.0.0-2000080, Metrelab Research).

**Gel Permeation Chromatography**: A Shimadzu GPC system with refractive index and diode array UV-Vis detector, run with THF solvent, was used. Samples are prepared at 5 mg/mL and filtered prior to analysis.

**Scanning Electron Microscopy**: Surface morphology was observed by scanning electron microscopy (JEOL JSM-7800 FLM) with an accelerating voltage of 5 kV and a working distance of 10-15 mm. Before observation, samples were coated with gold using a sputter coater (Desk II, Denton Vacuum Inc.).

**Small Angle X-ray Scattering**: A Rigaku Ultima IV Diffractometer acquired SAXS spectra from solid-phase samples. X-ray generation occurs within a 2.2 kW Cu K-alpha radiation long-fine focus tube (0.4 x 12 mm) with cross-beam optics. Scans are performed with a Theta/Theta wide angle goniometer from -3° to +154° (2θ) at 1 degree/minute. The signal is detected with a D/teX-ULTRA high-speed detector.

**Differential Scanning Calorimetry**: To determine materials' thermal and melting properties, a TA Instruments Discovery DSC was used according to protocols developed by the manufacturer with a scanning rate of 5°C/min. Each material was kept in aluminum pans, and an empty pan was used as the reference. All analyses were carried out in triplicate.

**Thermogravimetric Analysis:** To determine materials' thermal and melting properties, a TA Instruments Discovery TGA was used according to protocols developed by the manufacturer with a scanning rate of 20°C/min and with innert gas flow. All analyses were carried out in triplicate.

**Mechanical Testing**: Mechanical properties of scaffolds were measured using an MTS Synergie 200 mechanical tester (MTS Systems, Inc.) 15.0 mm in diameter x 3.0 mm thick scaffolds were prepared, n > 5 per composition and temperature. Compressive modulus was defined as the slope of the linear range on the resulting stress-strain curve, with a strain rate of 1.0 mm/minute.

**PLGA Nanoparticle Fabrication**: In the case of RhodamineB, a model drug: 15 mg of RhodamineB was dissolved in 500 µL of distilled water (ddH2O). Separately 250 mg of PLGA (50:50, MW = 7-17kDa, Sigma) was dissolved in 1.50 mL of DCM in a 50 mL Falcon Tube. Both solutions were kept on ice. Next, 160 µL of the drug solution was added to the PLGA solution and sonicated by a probe sonicator for 35 s on ice to create a w/o emulsion (35 V power). The w/o solution was poured into 5 mL of 1% w/v polyvinyl alcohol in distilled water and sonicated for 35 s on ice. The w/o/w emulsion was transferred to a 20 mL glass vial and stirred at 1300 rpm overnight in a fume hood to allow for solvent evaporation. The nanoparticles were concentrated and washed by six rounds of centrifugation at 9000 rpm for 20 min, each, refreshing diH2O between. Particles were reconstituted in ddH2O, lyophilized and stored at -20°C. In the case of simvastatin: a 38 mM stock solution was prepared in 1 mL ethanol and 1.5 mL 1 M NaOH (40 mg, 9.6 µmol), heated at 50°C for 2 hours until clear, and then neutralized to pH 7. Separately, 100 mg of PLGA (50:50, MW = 7-17kDa, Sigma) was dissolved in 1.51 mL of DCM in a 50 mL Falcon Tube. Next, 250 µL of the simvastatin solution was added to the PLGA solution and processed following the procedure described for RhodamineB. In the case of FITC-bovine serum albumin (BSA): a 1% w/v FITC-BSA solution was prepared in PBS. 225 mg of PLGA (50:50, MW = 7-17kDa, Sigma) was dissolved in 1.80 mL of DCM in a 50 mL Falcon Tube. Both solutions were kept on ice once fully dissolved, then 320 µL of the protein solution was added to the PLGA solution, and particles were fabricated as described.

**Evaluation of Drug-Release Kinetics**: Once fabricated, scaffolds were 8 mm diameter x 1.5 mm thick and incubated with PBS in a well plate at 37°C on an orbital shaker. Fluorescence spectroscopy was used to measure the concentration of released rhodamine B at various time points. PBS solution from each well was removed; the same volume of fresh PBS was added each time. A Thermo ScientificTM VarioskanTM LUX Multimode Microplate Reader was used to quantify the fluorescence.

**Histologic preparation of frozen sections**: PFA as exchanged for PBS for 24 hours at 4°C, then the PBS is exchanged for 30% sucrose for 4 days at 4°C. Samples are embedded in OCT for 4 hours at room temperature and then frozen. Serial sections (10-12 um thickness) are collected using a cryotome at -20°C.

**Histologic preparation of paraffin sections**: Samples prepared for frozen section were thawed and carefully removed from OCT, and OCT is exchanged for PBS over 10 days. Samples are dehydrated using an ethanol gradient and embedded in paraffin. Paraffin sections are cut at 5 um, stained following standard histologic preparations, and imaged with a bright field microscope (Olympus).

**Confocal laser microscopy:** A Nikon Eclipse C1 microscope is used for all confocal imaging. Frozen sections are thawed from -20°C and gently washed in PBS containing 0.1% Triton (PBST 0.1%) three times. Next, a 1:1000 solution of Hoescht nuclear stain was applied; the slides were washed three times in PBS then a coverslip was fixed with ProLong Gold Anti-Fade.

**CD31**: Histologic sections from paraffin were treated with xylene and an ethanol gradient to de-paraffinize. Antigen retrieval was performed by incubating 10 mM citrate buffer (pH 6.0). Sections were sequentially incubated in 5% BSA for 60 minutes and with primary antibodies (CD31, 1:100, Cell Signaling Technology, #3528) overnight at 4°C and DAB chromogen (Abcam, ab64261), mouthed with a coverslip, and imaged with a bright field microscope (Olympus).

**Mice:** The LysM-Cre mouse line was purchased from The Jackson Laboratories (Stock: 004781) and was on a C57BL/6 genetic background. These mice were bred with homozygous R26-tdTomato (RFP) reporter mice (B6.Cg-Gt(ROSA)26Sortm14(CAG-tdTomato)Hze/J, Jackson Lab, Stock #007914).
