# Supplementary figures and images for: Temperature-responsive PCL-PLLA nanofibrous tissue engineering scaffolds with memorized porous microstructure recovery
Source: Front Dent Med. Author manuscript; Available in PMC 2024 Apr 11. (PMC11008614; doi:10.3389/fdmed.2023.1240397)

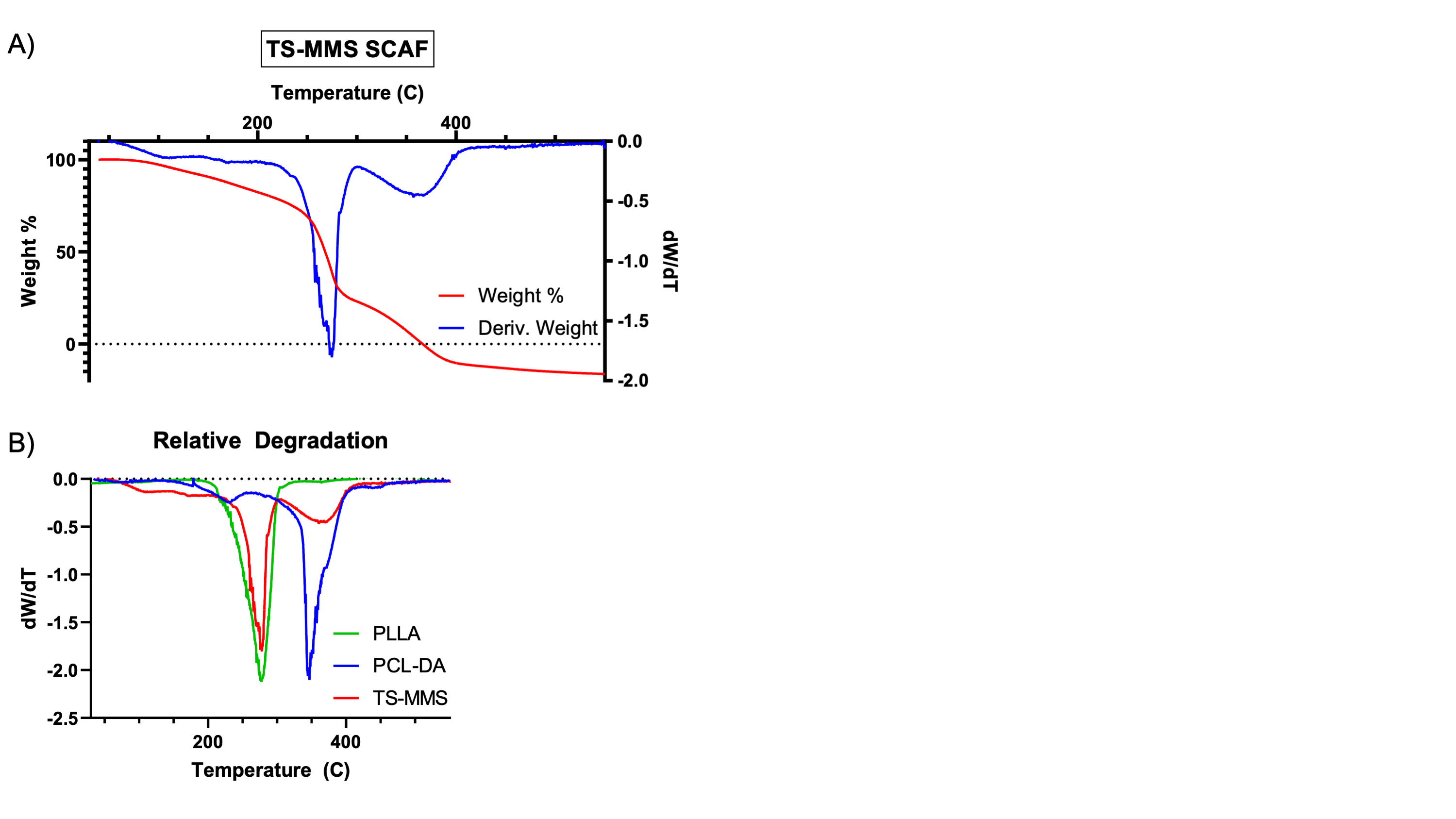

Supplement: Supplementary Figure 1 [file NIHMS1954334-supplement-Supplementary_Figure_1.tiff]
